# Anti‐metabotropic glutamate receptor 5 encephalitis: Five case reports and literature review

**DOI:** 10.1002/brb3.3003

**Published:** 2023-04-14

**Authors:** Sixian Chen, Haitao Ren, Fuhong Lin, Siyuan Fan, Yuze Cao, Weili Zhao, Hongzhi Guan, the Encephalitis Collaborative Group

**Affiliations:** ^1^ Department of Neurology, Peking Union Medical College Hospital Peking Union Medical College and Chinese Academy of Medical Sciences Beijing China; ^2^ Department of Neurology Affiliated Hospital of Chifeng University Chifeng Inner Mongolia China

**Keywords:** autoimmune encephalitis, herpes simplex virus, metabotropic glutamate receptor 5

## Abstract

**Objective:**

To describe the clinical and radiological characteristics of anti‐metabotropic glutamate receptor 5 (mGluR5) encephalitis.

**Methods:**

We reviewed the clinical data of five patients with anti‐mGluR5 encephalitis, and performed a literature review.

**Results:**

The five cases included a 52‐year‐old man who developed a biphasic course of anti‐mGluR5 encephalitis after herpes simplex encephalitis, a 22‐year‐old woman who showed bilateral basal ganglia lesions on brain magnetic resonance imaging (MRI), and a 36‐year‐old man with mixed aphasia and generalized tonic‐clonic seizures, a 51‐year‐old man presented with personality changes, hallucinations, delusions, sleeping disorders and a 58‐year‐old man with short‐term memory deficits and absence seizures.. There are 16 reported cases of anti‐mGluR5 encephalitis worldwide. Of all 21 patients, with a median onset age of 35 years old, the main neurological symptoms were cognitive impairment (85.7%, 18/21), psychiatric or behavior problems (76.2%, 16/21), seizures (57.1%, 12/21), sleeping disorders (52.4%, 11/21), different degrees of decreased consciousness (42.9%, 9/21), and movement disorders (23.8%, 5/21). Brain MRI was normal in 11 of 21 patients. Lesions of the limbic lobes were presented in 5 patients, while involvement of other extralimbic regions was also reported. Seven of 21 (33.3%) cases were combined with tumors. Elevated white blood cell counts or specific oligoclonal IgG bands in the cerebrospinal fluid were found in 18 of 21 patients, with marked improvements observed after immunotherapy.

**Discussion:**

Patients with anti‐mGluR5 encephalitis typically present with diffuse, rather than purely limbic, encephalitis. Anti‐mGluR5 encephalitis can be triggered by herpes simplex encephalitis. The risk of a combined tumor may be reduced in anti‐mGluR5 encephalitis patients.

## Graphical abstract

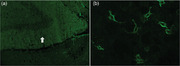

## 1 INTRODUCTION

Anti‐metabotropic glutamate receptor 5 (mGluR5) encephalitis is a rare neurological autoimmunity disease, which was first identified in two patients with limbic encephalitis (Ophelia syndrome) (Lancaster et al., 2011). With the increasing numbers of nonparaneoplastic cases, anti‐mGluR5 antibodies are now classified into intermediate‐risk antibodies using the latest diagnostic criteria for paraneoplastic neurologic syndromes (Graus et al., 2021).

To our knowledge, only 16 cases of anti‐mGluR5 encephalitis have been reported since the discovery of anti‐mGluR5 autoantibodies in 2011. In the present study, we report five additional patients with anti‐mGluR5 encephalitis from the Encephalitis Collaborative Group, including a case with post‐herpes simplex virus encephalitis (HSE) anti‐mGluR5 encephalitis, and a case with bilateral basal ganglia lesions on brain MRI. We also summarize the phenotype of this rare autoimmune encephalitis by reviewing peer‐reviewed cases reported in the English and Chinese literature.

## 2 METHODS

### 2.1 Patients and samples

From October 2018 to September 2022, paired serum and cerebrospinal fluid(CSF) samples from 260 patients with clinically suspected autoimmune encephalitis, but with negative common autoimmune encephalitis antibody panels (anti‐NMDAR/LGI‐1/GABAb‐R/CASPR2/GAD65/AMPAR; EUROIMMUN, Lübeck, Germany; FA 112d‐1005‐13 LS) and paraneoplastic antibody panels (anti‐Hu/Yo/Ri/CV2/Tr/Ma2/Amphiphysin; EUROIMMUN, Lübeck, Germany; DL 111‐1601‐2 G), were obtained and tested by the Neuroimmunology and Encephalitis Laboratory of Peking Union Medical College Hospital using a tissue‐based assay (TBA; EUROIMMUN, Lübeck, Germany). If specific patterns were shown in the TBA, additional antibodies, including the anti‐mGluR5 antibody (EUROIMMUN, Lübeck, Germany; FA 112n‐1005‐52 LS), were screened.

### 2.2 Literature review

Previous cases reported in English and Chinese peer‐reviewed journals between 1 January 1990 and 1 August 2022 were identified with the “PubMed/Medline,” “Web of Science,” “Embase,” and “China National Knowledge Infrastructure” databases, using the search strings “anti‐mGluR5 encephalitis” or “metabotropic glutamate receptor 5 AND encephalitis.”

### 2.3 Standard protocol approvals, registrations, and patient consents

This study was approved by the Ethics Committee of PUMCH (JS‐891). All patients or their representatives signed a written informed consent form.

## 3 RESULTS

### 3.1 Case 1

**FIGURE 1:**
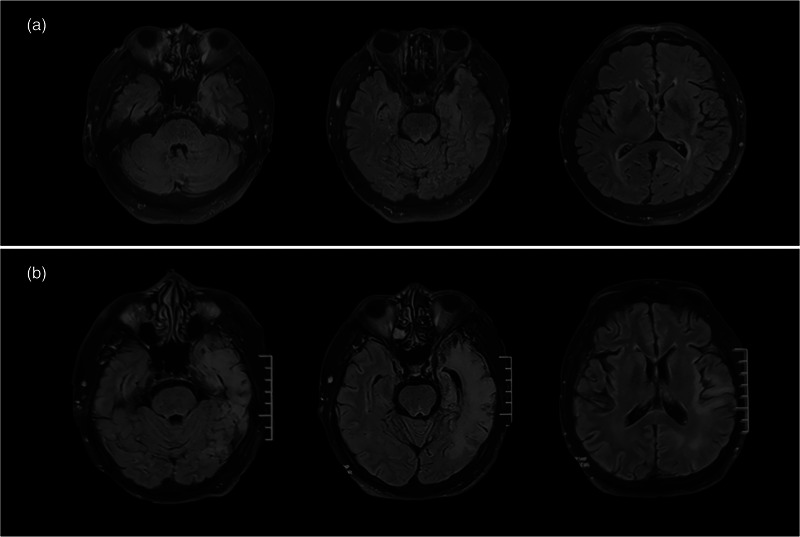
Brain Magnetic Resonance Imaging of Case 1. (a) Abnormal fluid‐attenuated inversion recovery(FLAIR) signals in the left medial temporal lobe at first admission of Case 1. (b) Expanded FLAIR hyperintensities in the left temporal, occipital, and insula lobes at second admission of Case 1.

A 52‐year‐old man was admitted to a hospital with fever, vomiting, diarrhea, generalized tonic‐clonic seizures, sensory aphasia. His Glasgow Coma Scale score at admission was E4V2M5. Brain MRI revealed hyperintense fluid‐attenuated inversion recovery (FLAIR) lesions in the left medial temporal lobe (Figure 1a). Electroencephalogram (EEG) showed synchronized high‐amplitude slow waves in the left frontotemporal regions. CSF showed 155 × 106/L leukocytes. Metagenomics next‐generation sequencing (mNGS) of the CSF was positive for herpes simplex virus‐1 (HSV‐1) DNA. The patient was treated with intravenous acyclovir, dexamethasone, and IV immunoglobulin (IVIg). One month later, his consciousness level was largely restored.

**FIGURE 2:**
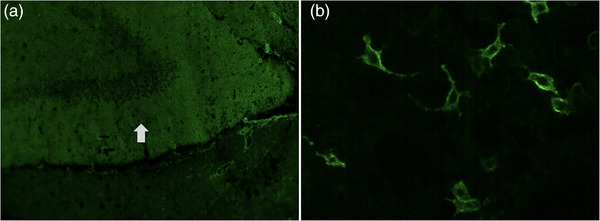
Examples of anti‐metabotropic glutamate receptor 5 antibodies in tissue‐based and cell‐based assays. The detection of autoimmune encephalitis autoantibodies in rat hippocampus sections was carried out using immunofluorescence and the results were then confirmed using a cell‐based assay that involved human embryonic kidney (HEK) 293 cells that over‐expressed the mGluR5 after they were transfected with the cognate cDNA. (a) Immunoreactivity of CSF from Case 1 in a tissue‐based assay, with neuropil (arrowed) staining of the hippocampus of rat brain sections (×100). (b) Reactivity of CSF from Case 1 in a cell‐based assay of HEK293 cells expressing mGluR5 (×200).

At 2 weeks after discharge, he developed a mental abnormality and exhibited decreased verbal output and frequent seizures. Neurological examination at re‐admission showed loss of time and place orientation. Brain MRI revealed FLAIR hyperintensities in the left temporal, occipital, and insula lobes (Figure 1b). EEG demonstrated continuous medium‐amplitude spikes in the left posterior temporal and occipital regions. CSF showed mild pleocytosis (19 × 106/L). Common autoimmune encephalitis panels were negative, while immunostaining of fixed rat brain sections with CSF showed a neuropil signal in the hippocampus (Figure 2a). Additional tests for rare autoimmune antibodies showed a positive anti‐mGluR5 antibody response using a cell‐based assay, with a titer of 1:100 (Figure 2b) in the CSF and 1:10 in the serum. Tumor screening was negative.

Diagnosed with post‐HSE anti‐mGluR5 encephalitis, he was administered intravenous methylprednisolone 40 mg/d for two weeks. At discharge, he could partially answer some daily questions related to time and place. At 1 month of follow‐up, he had mildly recovered from his severe mental disorder, with an modified Rankin Scale (mRS) of 4 points.

### 3.2 Case 2

**FIGURE 3:**
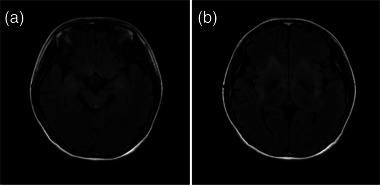
Brain magnetic resonance imaging of Case 2. Patchy fluid‐attenuated inversion recovery lesions were observed in the bilateral medial temporal lobes (a) and basal ganglia, and the bilateral insula lobes (b).

A previously healthy 22‐year‐old woman was hospitalized with insomnia and short‐term memory deficits for 1 month. At admission, she was in a state of somnolence. Her bilateral Babinski signs were positive. Montreal Cognitive Assessment score was 21/30. Head MRI revealed patchy FLAIR hyperintensities in the bilateral basal ganglia (Figure 3b), insula, and medial temporal lobes (Figure 3a). Screening tests for metabolic encephalopathy were negative. CSF demonstrated 7 × 106/L leukocytes. No infectious pathogen was identified in the mNGS of CSF. Common autoantibody panels for autoimmune encephalitis were negative while anti‐mGluR5 antibody tests were positive, with a titer of 1:10 in the CSF and 1:32 in the serum. The patient was prescribed intravenous methylprednisolone (80 mg/d) for 3 weeks, tapered to oral prednisone (50 mg/d). She was discharged with a markedly reduced level of drowsiness. At 5 months of follow‐up, she had largely returned to her normal life.

### 3.3 Case 3

A 36‐year‐old man was admitted because of mixed aphasia for 3 months and generalized tonic‐clonic seizures. Brain MRI showed no encephalitis‐related lesions. Spikes were noticed in bilateral frontal regions. CSF showed 2×106/L leukocytes, while specific OCB was positive. Anti‐mGluR5 antibody testing was positive in the serum (1:10) and negative in the CSF. Tumor screening was negative. Diagnosed with anti‐mGluR5 encephalitis, he was treated with glucocorticoids and IVIg. At 6 months of follow‐up, he was seizure free with significant improvement in his mixed aphasia.

### 3.4 Case 4

A 51‐year‐old man was admitted to the hospital with personality changes for 1 year, hallucinations, delusions and sleeping disorders for 3 months. Physical examination showed MoCA score was 7/30 (delayed memory 0/5). Abnormal signals were noticed in bilateral medial temporal lobes in brain MRI. CSF showed 16×106/L leukocytes. Anti‐mGluR5 antibody testing was negative in the CSF but positive in the serum (1:10). He was administered intravenous methylprednisolone (500 mg/d) for 5 days and tapered to oral prednisone (60 mg/d). At discharge, the score reached 13/30 and after 4 months of follow‐up, his mental state gradually became stable and he could even go out to buy food.

### 3.5 Case 5

A 58‐year‐old man presented with short‐term memory deficits and absence seizures for 1 year. Neurological physical examination was unremarkable. Brain MRI demonstrated hyperintense FLAIR lesions in the bilateral hippocampus and left insula lobe. EEG showed the spikes and slow waves primarily in the left anterior temporal and sphenoidal regions. There were 1×106/L leukocytes in the CSF and the anti‐mGluR5 antibody was negative in the CSF while positive in the serum (1:100). The patient was prescribed oral prednisone (60 mg/d) and 2 months later, the frequency of seizures reduced.

## 4 LITERATURE REVIEW

A total of 16 cases (12 in English and 4 in Chinese) of anti‐mGluR5 encephalitis have been previously reported (Table 1). Among all 21 cases, 13 were men (61.9%), with a median age of 35 years old. The main neurological symptoms were cognitive impairment (85.7%, 18/21) and psychiatric or behavioral problems (76.2%, 16/21), seizures (57.1%, 12/21), sleeping disorders (52.4%, 11/21), different degrees of decreased consciousness (42.9%, 9/21), and movement disorders (23.8%, 5/21). A wide range of cognitive domains were involved in the cognitive impairment, with speech dysfunction (23.8%, 5/21) and memory deficits (61.9%, 13/21) being the major manifestations.

**TABLE 1:** Clinical characteristics, treatment, and prognosis of 21 patients with anti‐metabotropic glutamate receptor 5 encephalitis

**Study**	**No./gender/age**	**Psychiatric symptoms**	**Cognitive dysfunction**	**Seizures**	**dLoC**	**Movement disorder**	**Sleeping disorder**	**Tumor**	**Limbic/extra—limbic lesions of MRI**	**CSF pleocytosis**	**Anti‐tumor/IVIg/steroids/PE/RTX**	**mRS at peak; last follow‐up (months)**
Lancaster et al. (2011)	No.1/F/46	+	+	+	–	+	–	HL	+/+	+	+/–/+/–/–	5;0 (48)
	No.2/M/15	+	+	+	–	–	–	HL	–/+	+	+/–/–/–/–	5;0 (72)
Mat et al. (2013)	No.3/M/35	+	+	–	–	–	–	HL	–/+	+	+/–/–/–/–	3;0 (38)
Pruss et al. (2014)	No.4/F/30	+	+	+	+	–	+	None	Normal	+	–/–/+/+/+	2;1 (48)
Spatola et al. (2018)	No.5/M/75	–	+	–	–	+	–	SCLC^a^	+/–	+	+/+/+/–/–	4;3 (62)
	No.6/F/40	+	+	–	+	+	+	None	Normal	+	–/–/+/+/–	5;0 (20)
	No.7/M/16	+	–	+	+	+	+	HL	Normal	+	+/–/+/+/–	4;0 (48)
	No.8/F/6	+	+	+	+	+	+	None	–/+	+	–/+/+/–/+	5;3 (19)
	No.9/F/20	+	+	–	+	–	+	None	Normal	+	–/–/–/–/–	4;0 (96)
	No.10/M/15	+	+	–	–	–	+	HL	Normal	+	+/+/+/–/–	4;2 (12)
	No.11/M/49	+	+	+	+	–	+	None	Normal	+	–/–/–/+/–/–	4;1 (5)
Guevara et al. (2018)	No.12/M/68	+	+	–	–	–	–	HL	–/+	– ; OCB	+/–/+/–/–	4;1 (1)
Chen et al. (2021)	No.13/M/51	+	–	+	+	–	–	None	Normal	+	–/+/+/–/+	3;6 (1)^b^
Guo et al. (2021)	No.14/M/32	+	+	–	–	–	+	None	Normal	– ; 24 h IgG↑	–/+/+/–/–	3;1 (12)
Liu et al. (2021)	No.15/F/12	–	+	+	–	–	–	None	Normal	– ; OCB	–/+/+/–/–	3;1 (3)
Feng et al. (2022)	No.16/F/22	–	–	+	–	–	+	None	Normal	–	–/+/–/–/–	2;1 (6)
This study	No.17/M/52	+	+	+	+	–	–	None	+/+	+	–/+/+/–/–/–	5;4 (1)
	No.18/F/22	+	+	–	+	–	+	None	–/+	+	–/–/+/–/–	3;1 (5)
	No.19/M/36	–	+	+	–	–	–	None	Normal	– ; OCB	–/+/+/–/–	3;1 (6)
	No.20/M/51	+	+	–	–	–	+	None	+/–	+	–/–/+/–/–	3;1 (4)
	No.21/M/58	–	+	+	–	–	–	None	+/–	–	–/–/+/–/–/–	2;1 (2)

Abbreviations: CSF = cerebrospinal fluid; dLoC = decreased level of consciousness; HL = Hodgkin's lymphoma; IVIg = IV immunoglobulin; MRI = magnetic resonance imaging; mRS = modified Rankin Scale; OCB = oligoclonal IgG bands; PE = plasma exchange; RTX = rituximab; SCLC = small cell lung cancer.

More detailed clinical manifestations include hallucinations in patients No.2/6/7/10/12/14/17/20; speech dysfunction in patients No.2/8/15/17/19; memory deficits in patient No.1/3/4/6/8/9/12/14/15/17/18/20/21.

^a^ This patient was also positive for the anti‐SOX1 antibody.

^b^ This patient was bedridden after tracheal intubation because of frequent seizures, resulting in pulmonary infection. He eventually died of respiratory failure and cardiac arrest.

Neuroimaging was normal in 52.4% (11/21) of patients. Lesions of the limbic lobes were present in 5 patients, while involvement of other extralimbic regions, including the cerebellum, thalamus, pons, frontal lobes, and parieto‐occipital cortex, were also reported. Seven of 21 (33.3%) patients had tumors, all but one of which were Hodgkin's lymphoma. Elevated CSF leukocytes were found in 15 of 21 patients, while OCB positivity was present in most of the remaining patients. The majority of patients treated with immunotherapy and/or anti‐tumor therapy achieved complete (mRS = 0; 28.6%, 6/21) or partial (a decrease in the mRS score ≥1 point; 66.7%, 14/21) remission at the last follow‐up.

## 5 DISCUSSION

Although some early studies reported anti‐mGluR5 encephalitis as limbic encephalitis (Abboud et al., 2021; Lancaster et al., 2011; Pruss et al., 2014), the more recent identification of a wide range of clinical symptoms (e.g., movement disorders) and extralimbic lesions by MRI indicate that this disorder is more similar to diffuse encephalitis. Bilateral basal ganglia lesions have not been previously reported in anti‐mGluR5 encephalitis. mGluR5s are widely expressed in the postsynaptic terminals, especially in the cerebral cortex, hippocampus and striatum of the basal ganglia (Abd‐Elrahman & Ferguson, 2022), which may explain the bilateral basal ganglia lesions in the present case. These findings provide further evidence that anti‐mGluR5 encephalitis fits the clinical classification of diffuse encephalitis (Leypoldt et al., 2015).

HSE is a potential trigger of autoimmune encephalitis. In previous studies, the most common type of post‐HSE autoimmune encephalitis was anti‐NMDAR encephalitis (Armangue et al., 2018). Additionally, other rare anti‐neuronal autoantibodies, including anti‐AMPAR antibodies, anti‐GABAb‐R antibodies, and anti‐dopamine‐2R antibodies, have been reported (Armangue et al., 2018; Mohammad et al., 2014).

To our knowledge, there are no other reports of anti‐mGluR5 encephalitis caused by HSV encephalitis. The mechanism of post‐HSE autoimmune encephalitis remains unclear. The prevailing pathogenic hypothesis of post‐HSE anti‐NMDAR encephalitis suggests that viral infection damages neurons and exposes NMDAR antigens located on the neuronal surface, initiating a series of autoimmune responses (Dalmau & Graus, 2018). Given that mGluR5s are widespread in the brain, a reduced density of mGluR5 clusters on the neuronal surface was noticed in anti‐mGluR5 encephalitis (Maudes et al., 2022), we speculate that the mechanism of post‐HSE anti‐mGluR5 encephalitis is similar to anti‐NMDAR encephalitis.

A positive anti‐mGluR5 antibody in the serum or CSF is a key diagnostic criterion for anti‐mGluR5 encephalitis. Currently, anti‐mGluR5 antibodies are not generally tested as frontline antibody assays for suspected autoimmune encephalitis. For highly suspected autoimmune encephalitis patients who are negative for common autoimmune encephalitis antibody panels and paraneoplastic antibody panels using cell‐based assays, screening for anti‐mGluR5 antibodies can be performed if specific staining patterns in the rat hippocampus neuropil are detected by TBA.

In addition to laboratory test results, the diagnosis of a certain type of encephalitis still requires a comprehensive evaluation of the clinical phenotype. Cases 3, 4, and 5 only showed positive results in their serum samples. These patients met the diagnostic criteria for encephalitis and their clinical phenotype was consistent with anti‐mGluR5 encephalitis and other possible autoimmune and viral encephalitis have been ruled out, so these patients were ultimately diagnosed with anti‐mGluR5 encephalitis. Moreover, some patients (Cases 3 and 4) underwent irregular immunotherapy at external hospitals before we obtained their serum and cerebrospinal fluid samples, which may also affect the results.

Although anti‐mGluR5 encephalitis was first reported as paraneoplastic autoimmune encephalitis, the increasing number of reported nontumor cases suggests that anti‐mGluR5 encephalitis may have a lower risk of a combined tumor(< 30%). In our five cases, there was no definite evidence of a combined tumor. Nevertheless, because neurological symptoms can appear 2–11 months earlier than tumor diagnosis (Spatola et al., 2018), these patients require screening for tumors at long‐term follow‐up.

A marked improvement was observed after immunotherapy in most reported anti‐mGluR5 encephalitis patients. However, in the present study, Case 1 had a poor outcome after IVIg and steroids, which may be due to the residual parenchymal damage after HSE. It was previously reported that the outcomes of post‐HSE autoimmune encephalitis patients were markedly worse than those of classical ones (Armangue et al., 2018), and that rituximab treatment could improve the residual symptoms in post‐HSE anti‐NMDAR encephalitis (Dorcet et al., 2020). Further studies examining the efficacy of rituximab in post‐viral autoimmune encephalitis are required.

In summary, we found that anti‐mGluR5 encephalitis is often associated with a wide range clinical symptoms and radiological extralimbic lesions and HSE is a potential trigger of anti‐mGluR5 encephalitis. Further reports of nonparaneoplastic patients indicate the lower risk of combined tumors in anti‐mGluR5 encephalitis.

## CONFLICT OF INTEREST STATEMENT

The author declares that there is no conflict of interest.

### PEER REVIEW

The peer review history for this article is available at https://publons.com/publon/10.1002/brb3.3003.

## Data Availability

Anonymized data are available from the corresponding author for replicating the procedures.
